# Genetic and environmental sources of behavioural individuality: a test of the standard model

**DOI:** 10.1098/rsbl.2026.0511

**Published:** 2026-10-28

**Authors:** Zachary J. Sweep, Raphaël Royauté, Ned A. Dochtermann

**Affiliations:** ^1^ Department of Biological Sciences, North Dakota State University, Fargo, ND, USA; ^2^ Environmental and Conservation Sciences Graduate Group, Fargo, ND, USA; ^3^ Department of Biological Sciences, INRAE, Paris, Île-de-France, France

**Keywords:** behavioural, personality, stochastic variation

## Abstract

Behavioural variation is typically assumed to arise from the combination and interaction of genetic and environmental variation. However, recent work with genetically identical individuals has found that substantial behavioural individuality is expressed even when genetic and environmental variation are negligible. This surprising result requires direct testing of our standard model for the sources of behavioural individuality. Here, we tested the standard model by comparing among-individual variation in highly inbred crickets versus outbred crickets. Comparing inbred and outbred lines allows for direct testing of the standard model by contrasting the magnitude of among-individual variation in a uniform versus varied genetic background. We found substantial and significant differences in among-individual variances, with among-individual variances being roughly three times greater in outbred versus inbred crickets (posterior probability, *p* = 0.974). Repeatability was also significantly different between inbred and outbred crickets (0.15 versus 0.41, respectively; *p* = 0.984). This result supports the standard model and suggests that the surprising expression of behavioural variation in clonal and parthenogenic species may represent an important but unique pathway for the expression of behavioural individuality.

## 1. Introduction

Understanding how variation in behaviours is distributed among individuals has been a central topic of behavioural research over the last 20 years [1–4]. Among-individual variation has been identified under a variety of labels, including animal personality [5,6], behavioural types [7] and behavioural individuality [8]. Regardless of the name used, behavioural individuality means that individuals differ from each other on average, even when each individual does not always show the exact same behavioural response from one time to the next [9]. A major finding of research into behavioural individuality is that among-individual variation is ubiquitous and accounts for approximately 40% of the variation observed within populations [1]. Both genetic and environmental sources with long-term impacts have also been identified as being major contributors to patterns of behavioural individuality (reviewed in [10]). However, recent research has suggested that our standard model of how behavioural individuality arises may be incorrect or, at least, incomplete.

Under the standard model, variation in behaviour emerges from the combination and interaction of genetic and environmental variation [10,11] and behavioural individuality represents the sum of genetic contributions and long-term environmental effects. This relationship between behavioural variability and sources of variation has been described in informal terms [3,4,12,13], in the context of quantitative genetics [10,11,13,14], and in statistical terms [15,16] for behavioural individuality and behavioural syndromes. This now represents the standard model for our understanding of behavioural individuality.

In this context, the relative magnitude of behavioural individuality is commonly estimated based on variance components as the adjusted repeatability [1,16–18]. Repeatability is the ratio of among-individual variance (*V_I_*) to total variance (*V_T_*), with *V_I_* being the genetic variance (*V_G_*) plus the variance due to what are known as permanent environmental effects (*V_PE_*; [10,19]):

$$\begin{array}{r} repeatbility=\frac{V_{I}}{V_{T}}=\frac{V_{G}+V_{PE}}{V_{G}+V_{PE}+V_{TE}+V_{e}}. \end{array}$$

*V_T_* includes among-individual variance (*V_I_*), the effects of within-individual plasticity in response to unmeasured temporary environmental variation (*V_TE_*) and measurement error (*V_e_*). By including both genetic and environmental variances in its calculation, repeatability connects to our classic model and has guided research into behavioural individuality.

The contributors to repeatability have multiple biological causes. For example, *V_PE_* includes developmental plasticity in response to variation in environments during development [20,21] as well as non-genetic parental effects [22]. *V_PE_* also includes environmental influences on behaviour that have effects that last for at least the duration of measurement. These influences could include otherwise temporary state differences, contributing to changes in repeatability over time. *V_TE_* includes reversible plastic responses to short-term environmental influences (i.e. phenotypic flexibility [23,24]). Both *V_PE_* and *V_TE_* include responses to shared and unshared environmental variation [21]. *V_G_* likewise combines multiple sources, including additive and dominance genetic variation and epistasis [21]. Mirroring issues in quantitative genetics, the standard model of behavioural individuality does not properly incorporate *G*×*E* effects.

Importantly, the standard model is potentially incomplete—or possibly outright incorrect—because phenotypic variance has been identified in multiple systems where both genetic and environmental variation are assumed to be approaching zero. This ‘intra-genotypic variation’ (IGV) has been identified in fruit flies [25–27], rodents [28], crustaceans [29], poecilid fish [30] and other taxa (reviewed by Bruijning *et al.* [31]). IGV has been argued to potentially be adaptive and suggestive of bet-hedging [32,33], is partially inducible [25,26] and there is genetic variation for its magnitude [26,27]. The presence of IGV challenges our standard model: if IGV manifests as *V_I__,_* then behavioural individuality may simply be an emergent property of developmental systems and neither genetic nor environmental variation is necessary.

The challenge that IGV poses for the standard model has most clearly been described in the context of work with Amazon mollies (*Poecilia formosa*)—a species that reproduces parthenogenically. In mollies, researchers have found that IGV generates among-individual variation in the absence of apparent environmental variation [8,34,35]. Moreover, the relative magnitude of this IGV is high enough that it suggests our standard model about the components of among-individual variation is incorrect [8]. In a parthenogenically reproducing species, like Amazon mollies, if individuals of the same genotype are reared under standardized laboratory conditions, the variance components *V_G_* and *V_PE_* should both approach zero. While *V_G_* will obviously approach zero in parthenogenic species, under standardized conditions the amount of environmental variation present should, by definition, be small and approaching zero. Consequently, repeatability should also approach zero. Contradicting this expectation, repeatability of activity behaviours in Amazon mollies was initially estimated as approximately 0.3 to 0.35 [8]. This is statistically indistinguishable from the mean of behavioural repeatability of 0.37, as reported in meta-analysis [1]. Subsequent research estimated repeatability of activity as ranging from approximately 0.2 to 0.7 under a variety of contexts [34–36]. These findings directly contradict our standard model and introduce the possibility that this model might be incomplete. As mentioned, these findings echo earlier reports of among-individual variation where researchers express there to be little [25–29]. For example, almost 30 years prior to the publication of repeatability estimates in mollies, Gartner [37] discussed the presence of random variation in phenotypes of lab rats (*Rattus norvegicus*). Specifically, Gartner [37] found that approximately 70% of the variation in highly inbred lab rat phenotypes was unexplained by known environmental variability. Gartner specifically advocated discarding the standard model. Given recent findings of substantial intra-genotypic behavioural variation and its persistence as an unexplained phenomenon, testing the generality of the standard model is of broad importance.

Here, we tested whether the standard model is sufficient to explain patterns of behavioural individuality in the banded cricket (*Gryllodes sigillatus*). To do so, we estimated the magnitude of *V_I_* in inbred and outbred lines of *G. sigillatus*. Our use of inbred lines is comparable to the systems in which the standard model has been challenged because the estimated relatedness within lines is between 0.995 and 1, and we similarly can control much of the environment, resulting in among-individual variances and repeatabilities that should approach 0. We can also determine whether the magnitude of variances is also lower in inbred versus outbred populations. This cannot be done with parthenogenically reproducing species. Importantly, because the standard model of behavioural individuality makes assumptions about the variance components summarized by repeatability, these variance components themselves must be estimated and compared.

## 2. Methods

To test the standard model, we measured the behaviour of individuals from five inbred and one outbred line of *G. sigillatus*. Inbred crickets were the product of 23 generations of full-sibling matings starting in 2001 [38]. This resulted in an inbreeding coefficient of approximately 0.99. Since then, for approximately 50 generations, mating was random within lines. This random mating will have led to an increased inbreeding coefficient due to drift and the stochastic loss of genetic variants. Consequently, individuals within lines can be considered effectively genetically identical.

Due to the lack of genetic variation within lines, inbred lines can be used to determine whether the standard model is sufficient. Rearing in a common environment allows for the assumption that *V_PE_* should approach zero. Since *V_G_* is also approaching zero in highly inbred lines, *V_I_* will approach zero. Under the standard model, we would therefore expect among-individual variation to be approaching zero in the inbred lines while being higher in the outbred line. The magnitude of *V_I_* can also be directly compared between inbred and outbred groups.

The outbred line was derived from individuals collected from Riverside, California, in 2014 [39]. The line was subsequently maintained with large population sizes and random mating. While this captive rearing necessarily resulted in some inbreeding, this line is nonetheless expected to have maintained greater genetic variation than is present in the inbred lines.

Inbred and outbred lines were reared and housed under shared conditions and in the same room, minimizing unshared environmental variation across lines. Lines were housed in large plastic containers (>40 l) and provided with ad libitum food and water. The standard lab diet, provided to all lines, had an average protein content of 16%. Lines were maintained under a reversed 12 h light : dark cycle and at 27°C.

### (a) Behavioural trials

We compared variation in exploratory propensity among and within lines. We measured exploratory propensity as activity level in an open field arena. All trials were conducted in an acrylic arena (60 × 60 cm and 15 cm high) with a Plexiglas lid. The arena was split into four 30 × 30 cm arenas separated by a divider, allowing four crickets to be tested at one time. We excluded trials in which an individual moved less than 5 cm; as in prior studies, this would have been classified as a lack of exploration [40–43]. This resulted in the exclusion of 67 observations from our eventual analysis.

Individual crickets were left to rest for 30 s under a 5‐cm‐diameter cup after being introduced into the lower right section of the arena (supplementary material, figure S1). After 30 s, we removed the cup, allowing individuals to move freely through the arena for 220 s. We estimated exploratory propensity as the distance travelled (centimetres) by the cricket using Ethovision X (Noldus Information Technology). This protocol has been used with *G. sigilattus* and other species of crickets [41,42,44,45].

Mass was recorded to the nearest milligram and temperature to one-tenth of a degree Celsius at the time of testing. Arenas were thoroughly cleaned between trials with 70% ethanol wipes to avoid accumulation of chemical traces of conspecifics. Individuals were measured for a maximum of three repetitions, with some individuals measured fewer times due to escape or natural mortality (table 1). Behavioural testing was conducted between June 2018 and May 2019 but, for logistical reasons (e.g. hatching and maturation times), testing was sequential across lines. In total, we included 611 behavioural trials across a total of 238 individuals in our analyses (table 1).

**Table 1.** Number of individuals and repetitions for each inbred line and from the outbred line.

	individuals × number of repetitions			
line	≥1	≥2	=3	total trials
A	40	38	26	104
E	34	26	18	78
F	40	36	23	99
G	40	31	24	95
H	34	30	24	88
outbred	50	50	47	147
total individuals	238	211	162	total: 611

### (b) Statistical analyses

Per the standard model for behavioural individuality, we predicted that *V_I_* would be effectively zero in inbred but not in outbred crickets. Relatedly, we predicted that, if not zero, among-individual variances in the inbred lines would be substantially lower than for outbred crickets. This stems from the possibility that even if environmental variation is sufficient to generate behavioural individuality in inbred crickets, the absence of genetic variation would still result in a lower among-individual variance. We were also interested in whether there were differences in behavioural averages and within-individual variances among lines but did not have *a priori* predictions about either. We estimated adjusted repeatabilities for inbred and outbred crickets for comparison to other studies and, to better understand the magnitude of the variances, we estimated coefficients of variation.

To determine whether among-individual variances differed between inbred and outbred crickets, we fit a mixed-effects model using the MCMCglmm package in R [46]. With this model, we allowed among- and within-individual variances to be estimated separately for inbred and outbred crickets. Individual cricket identity was fit as a random factor, and temperature during testing and mass were included as centred fixed effects. Following Royauté & Dochtermann [47], we evaluated the posterior distribution of the difference between among-individual variances between the outbred and inbred crickets. Within-individual variances were also allowed to differ between the inbred and outbred crickets. Variances were determined to be significantly different if >95% of posterior estimate differences were greater for one group of crickets versus the other (i.e. posterior probabilities). We then ran additional models where means and among- and within-individual variances were estimated separately by line. Markov chain Monte Carlo (MCMC) analyses were run for 1.3 × 10^7^ iterations with a 3 × 10^6^ burn-in and a 10 000-iteration sampling interval.

Means, variances, repeatabilities and coefficients of variation are reported as medians and 95% credibility intervals (CI) of posterior distributions [48]. Because of their magnitude, variances are reported as variance/10 000 to aid interpretability.

There are three main caveats to our research here. First, our analyses here represent exploratory tests of the standard model. The data were originally collected to address other questions and were opportunistically used here. Second, because of their different origins, the founding population of the inbred lines likely had a different starting magnitude of genetic variation. While this may have been higher or lower than that of the outbred population, the high level of inbreeding would nonetheless be expected to drive variances in the inbred lines to zero, while the random mating of the outbred line is expected to maintain variation. Third, there was an imbalance in our sample sizes, especially for our initial comparison of the outbred line and the pooled inbred lines. For this comparison, we had approximately 180 individuals across all the inbred lines and only around 50 individuals for the outbred line (table 1). However, three measurements of around 50 individuals are sufficient to estimate repeatabilities of 0.3 with high power and precision and are sufficient for precise estimation of within-individual variances [16]. Moreover, this lower sample size would increase estimated uncertainties and obscure differences, making testing more conservative.

## 3. Results

The estimated median of *V_I_* in open field distance for outbred crickets was 2.3 (95% CI: 1.1:4.3) versus 0.81 (0:1.6) for inbred crickets. As predicted by the standard model, *V_I_* was significantly greater in outbred crickets (figure 1A; outbred versus inbred median difference = 1.6 (0:3.6), 97.4% of posterior estimates positive). Across posterior estimates, *V_I_* of inbred crickets was approximately one-third that of the outbred crickets (0.34, 0:1.02).

**Figure 1.**
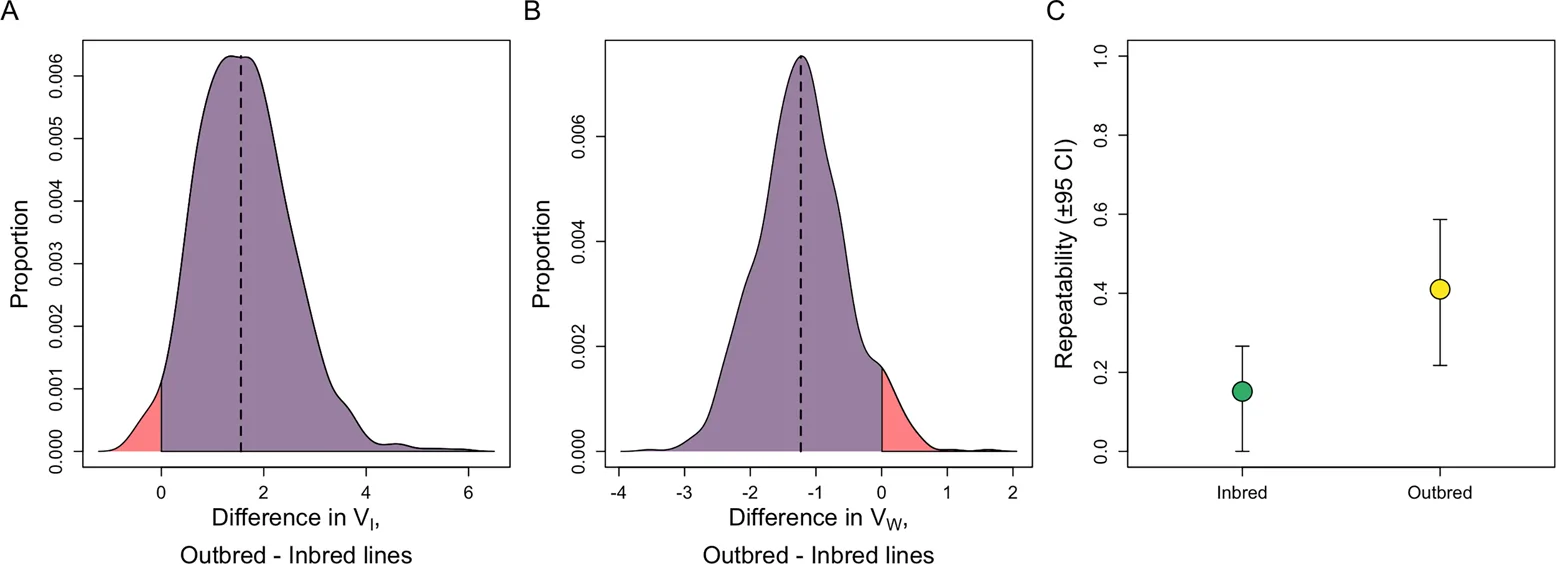
Variance differences and repeatability for inbred and outbred crickets. (A) MCMC posterior distribution of differences in among-individual variances (*V_I_*) between outbred and inbred crickets. The red portion of the distribution covers those estimates below zero. *V_I_* was estimated as being greater in outbred crickets for 97% of posterior estimates. (B) Posterior distribution of differences in within-individual variances (*V_W_*) between outbred and inbred crickets. The red portion covers those estimates above zero. *V_I_* was estimated as smaller in outbred crickets in 95% of posterior estimates. (C) Repeatabilities of activity in inbred (left) and outbred (right) crickets.

In contrast, the estimated within-individual variance was higher for inbred than outbred crickets. For outbred crickets, this variance was estimated as 3.4 (95% CI: 2.5:4.5) versus 4.6 (3.8:5.6) for the inbred crickets. The median difference estimated between inbred versus outbred crickets was −1.2 (−2.4:0.18), with within-individual variance for inbred crickets being greater in 95.2% of posterior estimates, indicating a significant difference between the groups (figure 1B). Across the posterior, the within-individual variance of outbred crickets was approximately three-quarters that of the inbred crickets (0.73, 0.53:1.05).

Combined, repeatability of open field distance was greater in outbred than inbred crickets (median difference: 0.27, 0.03:0.48). This difference was significant (98.4% of posterior estimates) and repeatability of the outbred crickets was 0.41 (0.22:0.59) versus 0.15 (0:0.27) for the inbred crickets (figure 1C).

Across lines, median distance travelled was highest in the outbred crickets (581.60 cm, 487.10:664.18; figure 2A), though one line of inbred crickets showed a similar degree of activity (line E: 519.95 cm, 369.93:649.22). Across lines, two of the inbred lines (E and H) had *V_I_*s that were roughly half that of the outbred crickets (figure 2B). The inbred lines A, F and G had *V_I_*s that were approaching zero (figure 2B). Within-individual variances differed across inbred lines but were generally of similar magnitude (figure 2C). Among-individual coefficients of variation were around 1% for three of the inbred lines but, as was the case for *V_I_*, higher in lines E and H (figure 3). Because of their higher *V_I_*, coefficients of variation for these lines were similar to the outbred line, though both had uncertainties that extended to zero.

**Figure 2.**
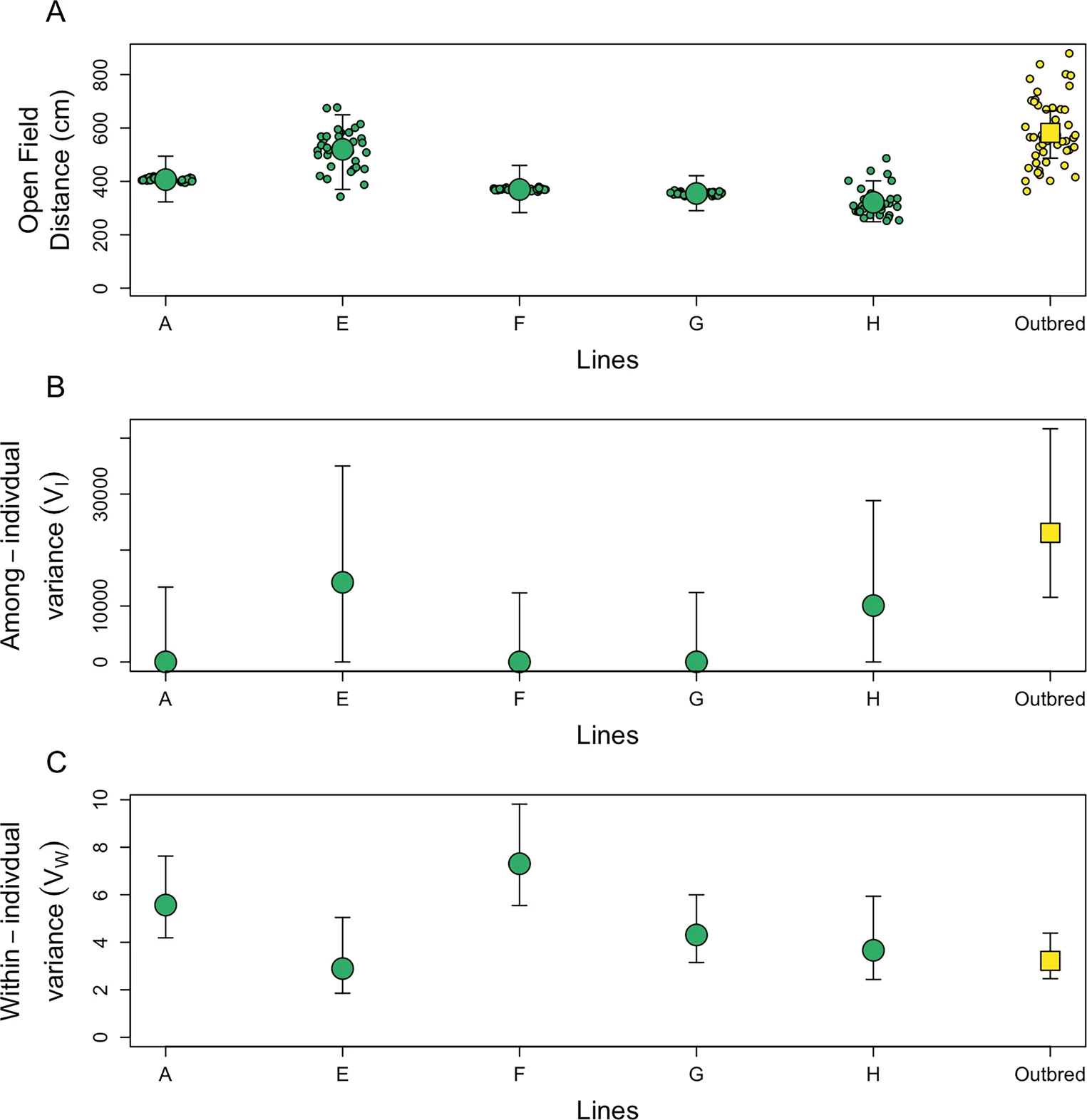
(A) Posterior median (large dots) and 95% CI (bars) of activity—as measured by distance travelled in an open field (centimetres)—for the inbred (green) and outbred line of crickets (yellow). Small points are estimated values for individual crickets (best linear unbiased predictors). (B,C) Median estimates (large dots) and 95% CI of among- and within-individual variances for the inbred (green) and outbred line (yellow) of crickets.

**Figure 3.**
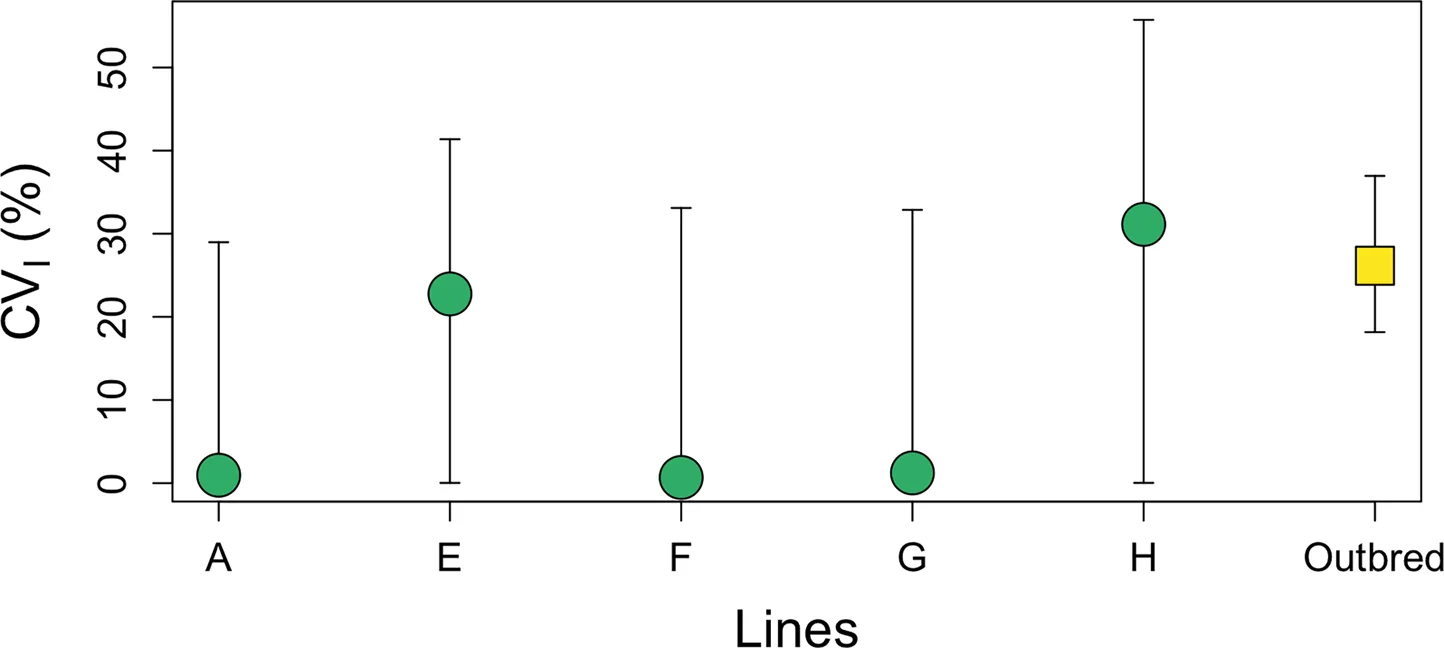
Posterior median estimates of coefficients of *V_I_* by line (as percentages) and 95% CI.

## 4. Discussion

We found that among-individual variation in outbred crickets was significantly greater than in inbred crickets (figure 1A). Likewise, repeatability in the inbred crickets was substantially and significantly lower than in the outbred crickets (figure 1C). These results largely support the standard model of behavioural individuality. This represents an important direct test of this model given recent observations that neither genetic nor known sources of environmental variation are necessary for behavioural individuality. While these sources of variation may not be necessary in parthenogenically reproducing systems, they were here. Put another way, while genetic and known environmental sources of variation may not be necessary in some systems, they were sufficient to generate among- and within-individual variation in the crickets studied here.

Interestingly, in two of our inbred lines (lines E and H), *V_I_* did not approach zero. Instead, variances were estimated as around half the magnitude estimated in the outbred group (figure 2B). This may be the case for multiple non-exclusive reasons. First, if introgression unknowingly occurred, inbreeding coefficients would be lower than expected, allowing for greater than expected *V_I_*. Second, if there is genetic variation in IGV, we would expect some lines to show greater among-individual variation. Third, since behavioural testing necessarily occurred in a disjunct manner across lines, microenvironmental effects may have differed by line and resulted in greater *V_I_* in some lines. We cannot conclusively distinguish among these explanations at this time.

The second explanation, that lines E and H may produce greater amounts of IGV, is perhaps the most biologically interesting and suggests genetic variation in IGV. Genetic variation in IGV has been observed in other species [27] and, in humans, loci associated with variability have also been identified [49]. Genetic variation in IGV fundamentally represents *G*×*E* effects, where the ‘*E*’ component is unknown and can include stochastic environmental effects or outcomes of stochastic processes (e.g. during development; reviewed by de Bivort [50]). Genetic variation in IGV has a number of interesting biological implications [31], especially regarding the potential for the evolution of bet-hedging strategies [26,33] and is an important topic for future research into behavioural individuality.

Consistent with microenvironmental effects explaining the higher *V_I_* in two lines, humidity in both the cricket housing and behavioural assay rooms is typically lowest during the winter (due to forced air heating). Line E, one of the high *V_I_* lines (figure 1D), was the only line measured during the winter. However, this explanation is not sufficient to explain the high *V_I_* estimated for line H, which was measured at the same time as line G. Generally, unmeasured or unmeasurable environmental variation can inflate among-individual variances when sampling is non-random [51,52]. While this could inflate differences between lines, it would not necessarily generate among-individual variation within lines. Nonetheless, the different lines were housed under standardized conditions, and there are no clear reasons to suspect this standardization is less than that in place for studies of IGV. Moreover, our work here differed from much of the existing research on IGV because we directly compared *V_I_* between groups. Importantly, IGV research that has focused on ratios like repeatability cannot differentiate between high magnitudes of *V_I_* and low *V_I_ and* low *V_T_* [47,53–55]. Finally, while IGV and microenvironmental explanations are interesting, and none of these explanations are mutually exclusive (either for our results or those of other authors), the more conservative and parsimonious explanation is that genetic introgression unknowingly occurred for lines E and H at some point.

We also found significant differences in within-individual variances between the inbred and outbred crickets. We did not have *a priori* predictions, and so we do not have a ready explanation for this finding. Relative to behavioural individuality, within-individual variation has been poorly studied despite its biological importance [56,57]. In crickets, increased within-individual variance may be indicative of greater behavioural flexibility and is higher when individuals have access to higher quality food sources [58]. Generally, given that within-individual variation largely stems from plasticity in response to temporary environmental variation [56,59], these findings support that even in controlled conditions individuals frequently experience different environments from each other. Future research into within-individual variation may better explain our findings here.

Recent behavioural research suggested that the standard model for behavioural individuality is insufficient [8,34,36]. These findings necessitated a reassessment and testing of our standard model. Our findings here indicate that the standard model may in some cases be sufficient, albeit not necessary. This conclusion suggests that findings elsewhere which supported a major role for IGV may instead be due to the specifics of an organism's reproductive biology, though—conflicting with this interpretation—IGV is increasingly recognized as common across taxa [25–31]. Whether due to bet-hedging resulting in higher fitness [33], inter-group selection (as may be the case with mutation rates; e.g. [60]), or other factors [61], mechanisms for the generation of variation may also have been selected for in systems showing high IGV. This is an intriguing possibility warranting further exploration. Regardless, the finding that our inbred crickets showed the expected and reduced among-individual variability supports the dominant assumptions of research regarding behavioural individuality.

## Supplementary Material



## Data Availability

Metadata, primary data, analysis code, code for figures and analysis output are publicly available at [62]. Supplementary material is available online [63].
