# Supplementary material for: Genetic and environmental sources of behavioural individuality: a test of the standard model
Source: Biol Lett. 2026 Oct 28;22(10):20260511. doi: 10.1098/rsbl.2026.0511 (PMC13725523; doi:10.1098/rsbl.2026.0511)
Supplement: Supplementary file 1 [file rsbl.2026.0511.zip › rsbl.2026.0511/rsbl20260511_si_001.docx]

Supplemental Materials:

Genetic and environmental sources of behavioral individuality: a test of the standard model

Zachary J. Sweep ^a,b^, Raphaël Royauté ^c^, and Ned A. Dochtermann ^a,d^

^a^ Department of Biological Sciences; North Dakota State University

^b^ Environmental and Conservation Sciences Graduate Group

^c^ Université Paris-Saclay, INRAE, AgroParisTech, UMR EcoSys,

91120 Palaiseau, France

^d^ corresponding author: [ned.dochtermann@gmail.com](mailto:ned.dochtermann@gmail.com)

Figure S1. Schematic of the behavioral testing arena. The red arrow represents a possible path, the length of which (in cms) would represent an individual’s activity score.
